# Clinical role of low hemoglobin ratio in poor neurologic outcomes in infants with traumatic intracranial hemorrhage

**DOI:** 10.1038/s41598-019-57334-6

**Published:** 2020-01-15

**Authors:** En-Pei Lee, Shao-Hsuan Hsia, Oi-Wa Chan, Chia-Ying Lin, Jainn-Jim Lin, Han-Ping Wu

**Affiliations:** 1 Division of Pediatric Critical Care Medicine, Department of Pediatrics, Chang Gung Memorial Hospital at Linko, Kweishan, Taoyuan, Taiwan; 2 College of Medicine, Chang Gung University, Taoyuan, Taiwan; 3 Department of Pediatric Emergency Medicine, Children’s Hospital, China Medical University, Taichung, Taiwan; 4 Department of Medical Research, Children’s Hospital, China Medical University, Taichung, Taiwan; 5 Department of Medicine, School of Medicine, China Medical University, Taichung, Taiwan

**Keywords:** Neonatal brain damage, Neonatal brain damage

## Abstract

Traumatic brain injury (TBI) is the leading cause of pediatric morbidity and mortality worldwide, and half of all fatalities occur in infants aged less than 1 year. We analyzed 129 infants diagnosed with TBI complicated with intracranial hemorrhage confirmed by brain computed tomography. We defined delta hemoglobin (ΔHB) as nadir HB - age specific mean HB, and the ratio of HB (%) as ΔHB/age specific mean HB x 100. Infants with poor neurologic outcomes had a lower admission HB and ΔHB (p < 0.05). The in-hospital mortality rate was 10.1% (13 infants), and the infants who died had a significantly lower ΔHB ratio compared to the survivors. The area under the receiving operating characteristic curve (AUC) of initial Glasgow Coma Score (GCS) in predicting neurologic outcomes was higher than that of ratio of ΔHB (0.881 v.s 0.859). In multivariate logistic regression analysis with the optimal cutoff ratio of ΔHB, it remained an independent predictor for in-hospital mortality and poor neurologic outcomes at discharge and at 6 months. AUC analysis for the ratio of ΔHB for poor neurologic outcomes in infants aged from 0–6 months was 0.85 and the optimal cutoff was −30.7% (sensitivity, 69%; specificity, 92%; positive likelihood ratio (LR^+^), 8.24; negative likelihood ratio (LR^−^), 0.34); the AUC was 0.88 in infants aged from 6–12 months and the optimal cutoff was −20.6% (sensitivity, 89%; specificity, 79%; LR^+^, 4.13; LR^−^, 0.15).

## Introduction

Traumatic brain injury (TBI) is the leading cause of pediatric morbidity and mortality worldwide and may be caused by child maltreatment. In the United States, more than 500,000 children suffer TBI each year, of whom 2,000 die^1^. Younger children are most at risk of TBI due to their dependency and inability to protect themselves, and half of all fatalities due to child abuse occurred in infants aged less than 1 year in 2015 in the United States^2^ with TBI being the major cause. Infants with TBI are often complicated with intracranial hemorrhage (ICH) due to high vascularization and physiologically more immature brains, which can cause anemia resulting in cerebral ischemic damage. In addition, the survivors of infant TBI frequently suffer from long-term poor neurologic outcomes^3,4^.

Anemia occurs when the number of red blood cells is not sufficient to meet the subject’s physiological needs and is the most common condition in all critical care settings, especially after TBI. The World Health Organization (WHO) defines anemia as a hemoglobin (HB) level <12 g/dl. An HB level <9 g/dl may be associated with poor outcomes and may indicate the need for a red blood cell transfusion (RBCT), however the benefits with regards to reducing morbidity and mortality are still unclear in adults^5^. Nevertheless, in order to maintain adequate oxygen delivery, liberal blood transfusions to maintain a HB level >10 g/dl is standard treatment^6,7^. For pediatric patients, the appropriate HB level differs according to age^8^, and guidelines for blood transfusions are limited^9^. Many recent studies have attempted to define the transfusion threshold for anemia in TBI, however the findings are controversial. To date, there is no golden standard threshold for blood transfusions in anemic adults or children^10,11^. The pathophysiology of anemia in TBI complicated with ICH is evidence of primary blood loss and decreased cerebral blood perfusion resulting in hypoxic damage^12^. Previous studies have reported that anemia is a risk factor for poor neurologic outcomes in adults with TBI complicated with ICH^13,14^. However, the role of anemia in pediatric TBI complicated with ICH has not been shown to be correlated with mortality^15^. Moreover, no study has focused on infants with TBI (aged <1 year) who account for most cases of pediatric TBI, and in whom TBI can cause cognitive and behavioral deficits in childhood resulting in a socio-economic burden on the families^16^. Therefore, the aim of this study was to assess the impact of anemia on neurologic outcomes and mortality in infants with TBI complicated with ICH.

## Materials and Methods

### Patient population

In this retrospective cohort study, we enrolled infants with TBI complicated with ICH admitted to the pediatric intensive care unit (PICU) and neurosurgical intensive care unit (NSICU) in a tertiary medical center from January 2006 to August 2017. The causes of TBI included assault, falls, traffic accidents, and being struck on the head accidently were based on the definition of the WHO^17^ for child maltreatment. The inclusion criteria were an age <1 year and TBI complicated with ICH confirmed by brain computed tomography (CT). Infants with TBI with internal bleeding or with other injured organs apart from intracranial bleeding were excluded. A total of 129 infants with TBI with ICH at admission were included in this study. This study was approved by the Institutional Review Board of Chang Gung Memorial Hospital in Taiwan. All methods were performed in accordance with the relevant guidelines and regulations. Data were collected, reviewed, de-identified, and anonymized before analysis, and the ethics committee waived the requirement for informed consent because of the anonymized nature of the data and scientific purpose of the study.

### Study design

Data including age (months), sex, Glasgow Coma Score (GCS), Injury Severity Score (ISS), reports of CT data, length of hospital stay, time of presentation (morning shift, night shift, graveyard shift), source of admission (emergency department, transfer from other hospitals), HB levels, neurologic outcomes, and mortality were obtained from both the social welfare reporting system and medical chart records. The infants with TBI were admitted to the PICU and NSICU following the Society of Critical Care Medicine guidelines^18^. Neurologic outcomes at discharge were evaluated by a pediatric neurologist using the Pediatric Cerebral Performance Category (PCPC) scale. Neurologic outcomes at 6 months after discharge were evaluated by clinicians at the outpatient department or via the telephone interview using the PCPC scale. Outcome scores were divided into two levels as: (1) favorable neurologic outcome (PCPC 1 to 2); and (2) poor neurologic outcome (PCPC 3 to 6 at discharge; PCPC 3 to 5 at 6 months after discharge).

### HB levels and transfusions

HB measurements during the hospital day included admission HB, nadir HB (lowest value during hospital stay), and mean HB (calculated from all values within 1 month). Daily or more frequent measurements of HB were performed if the initial HB was <9 g/dl or if there were any hypotensive episodes. Infants with an HB level <8.5 g/dl received a RBCT, however there is currently no gold standard for RBCTs in infants with ICH^19^. We used the following formulae because age-specific HB has been well documented in infants (Table 1), and we believed that these formulae could reflect the gap between anemia and age-specific HB more precisely:

$$\Delta {\rm{HB}}\,({\rm{g}}/{\rm{dl}})={\rm{nadir}}\,{\rm{HB}}-{\rm{age}}\,{\rm{specific}}\,{\rm{mean}}\,{\rm{HB}}.$$

$${\rm{Ratio}}\,{\rm{of}}\,{\rm{HB}}\,( \% )=\Delta {\rm{HB}}/{\rm{age}}\,{\rm{specific}}\,{\rm{mean}}\,{\rm{HB}}\times 100.$$

**Table 1:** Normal Hematologic Values during the First Year of Life in Healthy Infants.

Age (months)	0.5	1	2	4	6	9	12
Mean HB (g/dl)	16.6	13.9	11.2	12.2	12.6	12.7	12.7

From Saarinen UM, Slimes MA. Developmental changes in red blood cell counts and indices of infants after exclusion of iron deficiency by laboratory criteria and continuous iron supplementation. J Pediatr. 1978 Mar;92(3):412-6.

### Statistical analysis

The chi-square test or Fisher’s exact test was used to compare dichotomous variables between groups. Comparisons of continuous variables between two groups were made using the Mann-Whitney U test. Pearson’s correlation coefficients were used to compare correlations between important parameters. Logistic regression analysis was used to predict probabilities of poor neurologic outcomes (PCPC scale 3 to 6 at discharge and PCPC scale 3 to 5 at 6 months). Receiver operating characteristic (ROC) curves were used to determine the ideal cut-off values for HB for poor neurologic outcomes. The test characteristics of the different cut-off values, including sensitivity (Sn), specificity (Sp), area under the ROC curve (AUC), positive likelihood ratio (LR^+^), and negative likelihood ratio (LR^-^) were also examined. Statistical significance was defined at the p < 0.05 level, and all statistical analyses were conducted using IBM SPSS Statistics software (version 22.0; SPSS Inc., Chicago, IL, USA).

### Ethics approval and consent to participate

The study protocol was approved by the Institution Review Board and Ethics Committee of Chang-Gung Memorial hospital. IRB No.: 104–8307B.

## Results

### Participant characteristics

Table 2 shows the demographic information of the 129 infants. Fifty-two infants had favorable neurologic outcomes (PCPC 1 to 2), of whom 37 (71.2%) were male and the mean age was 6.6 ± 3.7 months (range 0.5 to 12 months). Seventy-seven infants had poor neurologic outcomes (PCPC 3 to 6), of whom 45 (58.4%) were male and the mean ± SD age was 5.3 ± 3.4 months (range 1 to 12 months). There were no significant differences in the source of admission and time of report between the favorable and poor neurologic outcome groups.

**Table 2:** Demographics and Neurologic Outcomes of Infants with Traumatic Intracranial Hemorrhage Admitted to the ICU.

Variables	All	Favorable	Poor	p-value
Patient number	129	52 (40.3)	77 (59.7)	
Age (months)	5.7 ± 3.5	6.6 ± 3.7	5.3 ± 3.4	0.038
Sex (male, n, %)	82 (63.6)	37 (71.2)	45 (58.4)	0.17
Source of admission (n, %)				0.119
Emergent department	49(38)	24(46.2)	25 (32.5)	
Transfer	80(62)	28(53.8)	52 (67.5)	
Time of report (n, %)				0.4
8:00~17:00	36(27.9)	17(32.7)	19 (24.7)	
17:00~24:00	52(40.3)	18(34.6)	34 (44.2)	
0:00~8:00	41(31.8)	17(32.7)	24 (31.2)	
Initial manifestations				
Initial GCS	11.5 ± 3.8	14.1 ± 2.1	9.8 ± 3.8	<0.01*
Initial ISS	25.3 ± 15.9	17.9 ± 5.1	30.3 ± 18.7	<0.01*
Retinal hemorrhage (n,%)	74 (57.4)	12 (23.1)	62 (80.5)	<0.01*
Laboratory and image findings				
Admission HB (g/dl)	9.5 ± 2	10.7 ± 1.7	8.8 ± 1.8	<0.01*
Mean HB (g/dl)	10.9 ± 7	10.9 ± 1.4	10.9 ± 9.1	0.97
Ratio of ΔHB (%)	−28.2 ± 15.7	−16.8 ± 12.7	−35.9 ± 12.6	<0.01*
SDH	105 (81.4)	35 (67.3)	70 (90.9)	<0.01*
EDH	17 (13.2)	14 (26.9)	3 (3.9)	<0.01*
SAH	43 (33.6)	10 (19.2)	33 (42.8)	<0.01*
IVH	17 (13.2)	2 (3.8)	15 (19.4)	0.015
DAI	24 (18.6)	3 (5.7)	21 (27.2)	0.013
Skull bone fracture (n,%)	32 (24.8)	19 (36.5)	13 (16.8)	0.012
Rotterdam CT score, median (IQR)	2 (2–3)	2 (2–2)	3 (2–4)	<0.01*
2 (n, %)	71 (55)	46 (88.4)	25 (32.5)	<0.01*
3 (n, %)	38 (29.4)	6 (11.6)	32 (41.5)	<0.01*
4 (n, %)	15 (11.6)	0	15 (19.5)	<0.01*
5 (n, %)	5 (4)	0	5 (6.5)	0.16
Treatments				
Acute neurosurgery (n, %)	73 (56.6)	19 (36.5)	54 (70.1)	<0.01*
Burr hole drainage	57 (44.1)	13 (25)	44 (57.1)	<0.01*
External ventricular drainage	6 (4.6)	2 (3.8)	4 (5.2)	0.72
Intracranial pressure monitor	12 (9.3)	0	12 (15.5)	<0.01*
Craniotomy and hematoma evacuation	13 (10.1)	7 (13.4)	6 (7.8)	0.45
Decompressive craniectomy	1 (0.7)	0	1 (1.3)	0.84
Mechanical ventilation (n, %)	53 (44.2)	6 (11.5)	47 (61)	<0.01*
RBC transfusion (n, %)	64 (49.6)	8 (15.3)	56 (72.7)	<0.01*
Outcomes				
	21 (9–25)	11 (7–13)	28 (15–31)	<0.01*
ICU LOS (days)	14 (4–17)	6 (3–8)	19 (8–23)	<0.01*

Good: PCPC 1 to 2; Poor: PCPC 3 to 6.

Results are presented as median (IQR), mean ± SD, or number (percent).

*p < 0.05 statistically significant; PCPC = Pediatric Cerebral Performance Category; ICU = intensive care unit; LOS = length of stay; SDH = subdural hemorrhage; EDH = epidural hemorrhage; SAH = subarachnoid hemorrhage; IVH = intraventricular hemorrhage; HB = hemoglobin; DAI = diffuse axonal injury; RBC = red blood cell.

**Figure 1:**
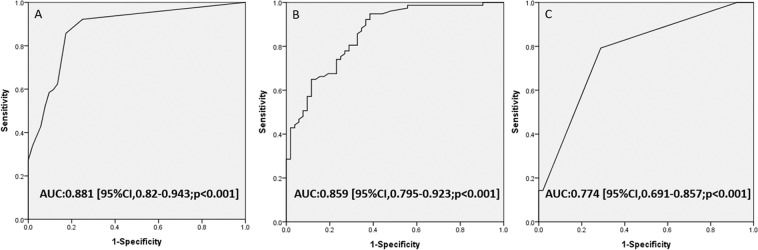
Receiver operating characteristic curves to assess the predictive accuracy of GCS, ratio of ΔHB (%) and ISS for neurologic outcomes at discharge. (**A**) Initial GCS. (**B**) Ratio of ΔHB (%). (**C**) ISS.

The initial manifestations were more severe in the poor neurologic outcome group, with a significantly lower initial GCS and significantly higher ISS (p < 0.05). In addition, the admission HB level and ratio of ΔHB were lower and the number of RBCTs were higher in the poor neurologic group (p < 0.05). The mean time to detect the ratio of ΔHB was 3.6 ± 3.5 hours after admission. The poor neurologic group had a significantly higher Rotterdam CT score (p < 0.05), and also significantly higher rates of retinal hemorrhage, need for neurosurgery and mechanical ventilation (p < 0.05). Moreover, the poor neurologic group had longer lengths of stay in the hospital and ICU (*p* < 0.05). The in-hospital mortality rate was 10.1% (13 infants) (Table 3). In univariate analysis, initial GCS, ISS, ratio of ΔHB and Rotterdam CT score were associated with in-hospital mortality. The correlations among the three parameters (GCS, ISS, and ratio of ΔHB) were statistically significant. Initial GCS was positively correlated with ratio of ΔHB (r = 0.444, p < 0.001) and negatively correlated with ISS (r = −0.553, p < 0.001). The ratio of ΔHB was negatively correlated with ISS (r = −0.346, p < 0.001). The ROC curves to assess the predictive accuracy of GCS, ISS and ratio of ΔHB in predicting neurologic outcomes at discharge are shown in Fig. 1. The AUCs were 0.881 for initial GCS, 0.859 for the ratio of ΔHB, and 0.774 for ISS (Fig. 1).

**Table 3:** Univariate Analysis of Factors Associated with In-hospital Mortality.

Variables	Survivors	Died	p-value
Patient number	116	13	
Age (months)	5.9 ± 3.6	4.1 ± 3.2	0.071
Sex (male, n, %)	75 (64.6)	7 (53.8)	0.64
Initial GCS	12.3 ± 3.2	4.7 ± 2.8	<0.01*
Initial ISS	20.6 ± 5.1	67.3 ± 18.8	<0.01*
Retinal hemorrhage (n, %)	63 (54.3)	11 (84.6)	0.072
Admission HB (g/dl)	9.6 ± 1.9	8.5 ± 2.5	0.098
Mean HB (g/dl)	11 ± 7.4	9.8 ± 1.5	0.751
Ratio of ΔHB (%)	−26.8 ± 15.3	−41.1 ± 13.5	<0.01*
Rotterdam CT score, median (IQR)	2 (2–3)	4 (3–5)	<0.01*
Neurosurgery (n, %)	68 (58.6)	5 (38.4)	0.27
RBC transfusion (n, %)	54 (46.5)	10 (76.9)	0.074

Results are presented as median (IQR), mean ± SD, or number (percent).

*p < 0.05 statistically significant; GCS = Glasgow Coma Scale; ISS = injury severity score; HB = hemoglobin; CT = computed tomography; RBC = red blood cell.

### Association between HB and other risk factors and poor outcomes

**Figure 2:**
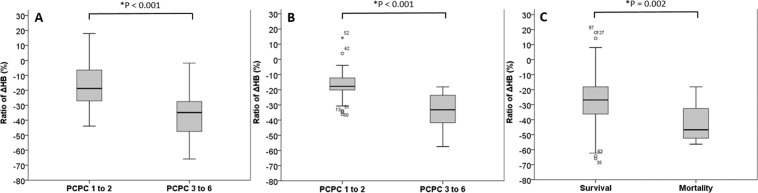
Ratio of ΔHB (%) and outcome at discharge. The ratios of ΔHB (%) ± SD were: (**A**) −16.9 ± 14.7 vs −37.1 ± 13.1 for favorable and poor outcomes in the 0–6 month-old infants. (**B**) −16.8 ± 11 vs −33.6 ± 11.3 for favorable and poor outcomes in the 6–12 month-old infants. (**C**), −26.8 ± 15.3 vs −41.1 ± 13.5 for those who survived and those who died in the 0–12 month-old infants.

**Figure 3:**
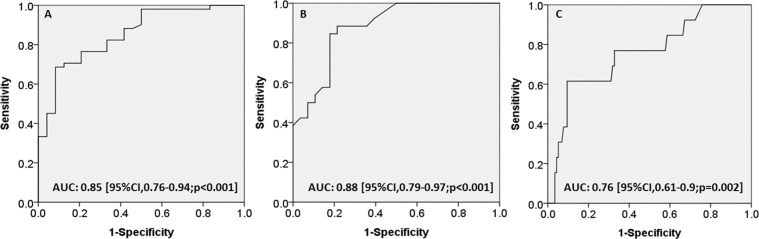
Receiver operating characteristic curves to assess the predictive accuracy of the ratio of ΔHB (%) for outcomes at discharge. (**A**) 0–6 month-old infants for poor neurologic outcomes. (**B**) 6–12 month-old infants for poor neurologic outcomes. (**C**) 0–12 month-old infants for in-hospital mortality.

The ratios of ΔHB were significantly lower in the favorable neurologic outcome group compared to the poor neurologic group at discharge in the infants aged 0–6 months (Fig. 2A) and in those aged 6–12 months (Fig. 2B) (both *p* < 0.05), and significantly lower in the survivors compared to those who died (Fig. 2C) (*p* < 0.05). The AUC for the ratio of ΔHB for poor neurologic outcomes in the infants aged 0–6 months 0.85 (95% CI, 0.76–0.94, *p* < 0.05), and the optimal cutoff value was −30.7% (Sn, 69%; Sp, 92%; LR^+^, 8.24; LR^−^, 0.34) (Fig. 3); the AUC was 0.88 in the infants aged 6–12 months (95% CI, 0.79–97, *p* < 0.05), and the optimal cutoff value was −20.6% (Sn, 89%; Sp, 79%; LR^+^, 4.13; LR^−^, 0.15) (Table 4). In addition, for predicting mortality, the AUC was 0.76 (95% CI, 0.61–0.9, *p* < 0.05) (Fig. 3C) and the optimal cutoff value was −46.4% (Sn, 62%; Sp, 89%; LR^+^, 5.95; LR^−^, 0.43) for all infants. We further performed multivariate logistic regression analysis to predict in-hospital mortality and a poor neurologic outcome (PCPC ≥ 3) at discharge and 6 months after discharge, including sex, initial GCS, ISS, ratio of ΔHB, RBCT, imaging findings, retinal hemorrhage, neurosurgery, and skull bone fractures (Table 5). In the final model, only initial GCS and ratio of ΔHB remained significant predictors for mortality and poor neurologic outcomes at discharge and after 6 months.

**Table 4:** Best Predictive Power of the Ratio of ΔHB (%) for Outcomes at Discharge.

Outcome	Age Group (months)	Ratio of ΔHB (%)	Sensitivity	Specificity	LR^+^	LR^−^
Poor neurologic	0–6 (n = 75)	−30.7%	0.69	0.92	8.24	0.34
outcomes	6–12 (n = 54)	−20.6%	0.89	0.79	4.13	0.15
Mortality	0–12 (n = 13)	−46.4%	0.62	0.89	5.95	0.43

LR + = positive likelihood ratio; LR- = negative likelihood ratio

**Table 5:** Multivariate Logistic Regression Analysis to Predict Outcomes (PCPC ≥3) at Discharge and 6 months.

Outcome	Age Group (months)	Parameters	Discharge		6 months after discharge	
			Adjusted odds ratio	p-value	Adjusted odds ratio	p-value
Poor neurologic outcomes	0–6	Initial GCS (3–8 vs 9–15)	90.6 (9.9–832.5)	<0.001	28.6 (4.3–191.9)	0.001*
		Ratio of ΔHB (%) (<−30.7% vs >−30.7%)	40.2 (3.5–461.4)	0.003*	15.4 (2.8–83.3)	0.001*
	6–12	Initial GCS (3–8 vs 9–15)	59.3 (5.1–687.1)	0.001*	30.2 (2.7–343.7)	0.006
		Ratio of ΔHB (%) (<−20.6% vs >−20.6%)	21.8 (3.1–153.3)	0.002*	45.1 (4.2–486.7)	0.002*
In-hospital mortality	0–12	Initial GCS (3–8 vs 9–15)	13.9 (2.9–66.6)	0.001*		
		Ratio of ΔHB (%) (<−46.4% vs >−46.4%)	14.4 (3.1–68.3)	0.001*		

The adjusted odds ratios were obtained from a multivariate logistic model which included sex, initial GCS, ISS >15, red blood cell transfusion, imaging findings, retinal hemorrhage, neurosurgery, and skull bone fractures; *p < 0.05 statistically significant; GCS = Glasgow Coma Scale; ISS = injury severity score; HB = hemoglobin.

## Discussion

In this study, we demonstrated that a lower HB level was an independent risk factor for poor neurologic outcomes and in-hospital mortality in infants with TBI complicated with ICH. In addition, a lower HB level during admission was more common in the infants with TBI with poor neurologic outcomes compared to those with favorable neurologic outcomes. We also found that a lower HB was associated with poor neurologic outcomes and in-hospital mortality independent of other risk factors including initial GCS, ISS, RBCT, neurosurgery, imaging findings, and retinal hemorrhage, all of which have been reported to be strong predictors for outcomes in infants with TBI in previous studies^15,20,21^.

Previous studies have reported that initial GCS and ISS are both risk factors for a poor prognosis in pediatric TBI cases^22,23^. In the current study, we found that initial GCS, ISS and ratio of ΔHB were statistically significantly correlated. In addition, we showed that the infants with poor outcomes had lower GCS, higher ISS and lower ratio of ΔHB. A change in ΔHB may indicate changes in the severity of systemic injuries, and the ISS and GCS may be affected by these changes. Previous studies have also reported that the pathophysiology of low HB is correlated with poor neurologic outcomes in patients with TBI through two potential mechanisms. First, a decrease in HB has been shown to be proportional to the volume of primary blood loss, and that this is a powerful predictor for a poor prognosis^12,24^. Second, low HB can result in secondary brain injury due to cerebral hypoxia. Cerebral oxygen delivery (DO2) is calculated as cerebral blood flow (CBF) multiplied by arterial oxygen content (CaO2). More oxygenation is needed in an injured brain, however impaired global cerebrovascular autoregulation can cause compensatory mechanisms to fail^7,12^. Consequently, there is increased CBF in patients with an injured brain, however this is still insufficient to compensate for the reduction in CaO_2_ induced by the low HB. Therefore, a decrease in cerebral DO2 develops and results in cerebral hypoxia. This may explain why a decrease in HB has a detrimental effect on neurologic outcomes in infants with TBI.

Infants agedv <1 year may have different normal HB levels, and the values of mean HB on admission may not reflect the real severity of anemia. Therefore, we defined the ratio of ΔHB on the basis of nadir HB to identify the real gap in age-specific standard HB, and reflect the worst condition of anemia. We hypothesized that the nadir HB may reflect the proportion of primary blood loss and the lowest CaO2, thereby indicating the severity of cerebral hypoxia. In addition, the nadir HB developed within the first 2 days, and was thus a feasible and practical parameter. In previous studies on pediatric TBI, initial GCS and ISS were the most commonly used risk factors for a poor prognosis^20–23^. In the final model of our study, we identified initial GCS and ratio of ΔHB as the predictors for a poor prognosis, both of which were more powerful than ISS. Our study demonstrated that the AUC of GCS in predicting neurologic outcomes was higher than that of ΔHB ratio. This may indicate that initial GCS could show higher predictive power for neurologic outcomes than ratio of ΔHB. In fact, initial GCS is a well-known objective scale to represent the initial severity of TBI and is also a good predictor for neurologic outcomes. However, the trend of ratio of ΔHB could provide information about disease severity and progression. Our results suggest that if an initial brain CT and GCS are not critical and do not indicate aggressive interventions, a lower ratio of ΔHB may serve as a parameter to assess disease progression and the need for further interventions. Furthermore, this is the first study to show that the ratio of ΔHB can be as powerful a predictor as initial GCS for in-hospital mortality. The cutoff value for the ratio of ΔHB for a poor neurologic outcome was lower in the infants aged 0–6 months compared to those aged 6–12 months. This indicates that 0–6 month-old infants may have a higher proportion of blood loss than 6–12 month-old infants, and that this leads to poor neurologic outcomes. The may be due to the followings reasons. First, the fontanelle is bigger and the sutures of skull bones are not tight in 0–6 month-old infants, so a higher proportion of blood loss may be required to cause an increase in intracranial pressure thereby resulting in a poor prognosis. Second, in normal growth milestones, 0–6 month-old infants are less active than 6–12 month-old infants, and therefore the caregivers may ignore the clinical manifestations in 0–6 month-old infants with TBI such as drowsiness or poor feeding, resulting in a delayed diagnosis and greater blood loss. The cutoff value was the lowest in those who died in the hospital, indicating greater blood loss. Greater blood loss in the brain can cause a bigger mass effect, higher intracranial pressure, and hypotension, resulting in severe cerebral hypoxia. All of these factors may be correlated with mortality. Previous studies have reported that RBCTs can be benefit, harmful or have no impact on neurologic outcomes in patients with TBI^25–30^. In the current study, about half of the cohort (64 infants, 49.6%) received RBCTs, however RBCTs were not an independent predictor for a poor prognosis in the multivariate model. Prospective randomized trials to evaluate the intervention in pediatric traumatic ICH may be necessary to clarify this issue.

## Conclusions

The current study demonstrated that a lower HB level was associated with in-hospital mortality and poor neurologic outcomes in infants with TBI, which is consistent with previous reports in adults but has not been reported in children. Furthermore, we identified that the best cutoff value of the ratio of ΔHB for outcomes was −30.7% in 0–6 month-old infants and −20.6% in 6–12 month-old infants for poor neurologic outcomes, and −46.4% for in-hospital mortality. Further randomized control trials are needed to investigate the impact of RBCTs based on these cutoff values.

## Data Availability

The datasets used and analyzed during the current study are not publicly available because of the Child Protection Medical Service Demonstration Center regulations, however they are available from the corresponding author on reasonable request. The study was supported in part by the China Medical University Hospital (DMR-108–198), Research Laboratory of Pediatrics, Children’s Hospital, China Medical University.
